# Retraction: A Cautionary Tale of a Complex Peri-Trochanteric Fracture in a Very Important Person (VIP) Patient at a Community-Based Hospital: A Case Report

**DOI:** 10.7759/cureus.r66

**Published:** 2023-01-19

**Authors:** Jason P Den Haese, Blake E Delgadillo, Bryan G Anderson, Shawn W Storm

**Affiliations:** 1 Department of Orthopedic Surgery, Lake Erie College of Osteopathic Medicine (LECOM) Health, Millcreek Community Hospital, Erie, USA; 2 Department of Orthopedic Surgery, Lake Erie College of Osteopathic Medicine (LECOM), Bradenton, USA; 3 Department of Spine Surgery, Swedish Neuroscience Institute, Seattle, USA

This article has been retracted due to procedural errors, specifically as they relate to the relevant institution’s research review process, the use of internal morbidity & mortality cases, and the failure to properly notify the physician of record. The journal was contacted with these concerns by the surgeon of record, who was not included as an author despite being the lead surgeon on this case. The surgeon of record and Dr. Anderson and Dr. Storm met to discuss the situation and agreed that the article must be retracted. They provided the following statement: 

"We agree that the article was written in a conjectural manner, making assumptions about aspects of the clinical decision-making that could only be known by the surgeon of record (the one making those decisions), to fit a narrative about a perceived “VIP status” affecting the patient’s care. This accusation was entirely unfounded, but was believed to add impact to the paper. Dr. Anderson was the only author with knowledge of the patient case and had a responsibility to publish a high-quality manuscript with correct information. Dr. Anderson initially reviewed the paper and felt the “VIP” piece of the paper was not appropriate and advised its removal from the manuscript. However, he failed to review the paper in its final form and gave permission for the principal author, Dr. Jason Den Haese, to seek publication. As the senior resident and only author with knowledge of the patient in this case report, Dr. Anderson was responsible for ensuring the final publication was accurate in its contents, and he did not review the paper prior to final submission. Dr. Anderson wholeheartedly agrees that he neglected his responsibilities as an author. Moreover, Dr. Anderson was complicit in publishing a paper that made arguments regarding a surgeon and then did not discuss those arguments with the very surgeon they involved."

